# Supplementary material for: Yin Yang 1 sustains biosynthetic demands during brain development in a stage-specific manner
Source: Nat Commun. 2019 May 16;10:2192. doi: 10.1038/s41467-019-09823-5 (PMC6522535; doi:10.1038/s41467-019-09823-5)
Supplement: Supplementary file 1 — Supplementary Information [file 41467_2019_9823_MOESM1_ESM.pdf]

SUPPLEMENTARY INFORMATION

Yin Yang 1 sustains biosynthetic demands during brain development in a stage-specific manner.

Zurkirchen et al.

Supplementary Figure 1

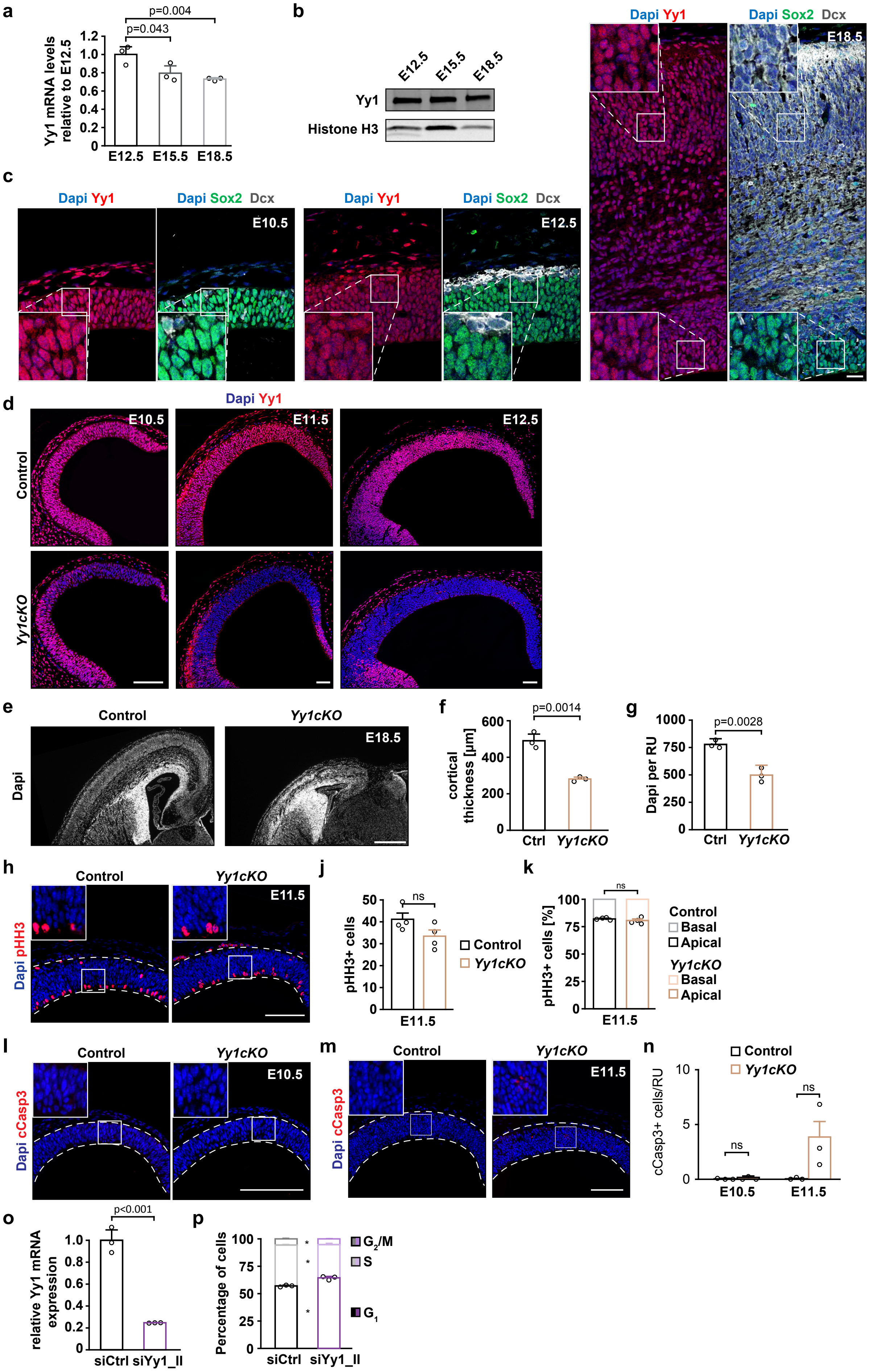

Supplementary Figure 1. Yy1 regulates cortex development.

a) qRT-PCR for Yy1 mRNA levels at different stages of cortex development.

b) Immunoblot depicting Yy1 protein levels at different stages of cortex development.

c) Immunohistochemical analysis of Yy1 expression pattern during cortex development. Yy1 is expressed in Sox2-positive neural progenitor cells and doublecortin-positive (Dcx) immature neurons at all embryonic stages.

d) Efficient ablation of *Yy1* in the dorsal cortex of *Yy1cKO* embryos. Single cells start losing Yy1 expression from E10.5 on. By E11.5, virtually all cells of the dorsal cortex have lost Yy1 protein expression.

e-g) *Yy1cKO* embryos exhibit decreased cortical thickness and less cells (Dapi) per radial unit (RU = 100 µm) compared to control embryos at E18.5.

h-k) Immunohistochemistry for pHH3 shows that the number of mitotic cells starts to decrease, but is not yet statistically significant, in *Yy1cKO* embryos at E11.5. The ratio of apical vs. basal pHH3+ does not change **(k)**.

l-n) Immunohistochemistry for cCasp3 shows that the number of apoptotic cells starts to increase, but is not yet statistically significant, in *Yy1cKO* embryos at E11.5.

o,p) Knockdown of Yy1 by a second siRNA efficiently reduces mRNA levels and induces G1/S cell cycle arrest as assessed by flow cytometry.

Nuclei are counterstained with Dapi. Scale bars represent 400 µm **(e)**, 100 µm **(h,l,m)**, 50 µm **(d)**, 20 µm **(c)**. Comparisons were performed using the 2-tailed unpaired Student's t-test. Data are the mean ± standard deviation.* $p < 0.05$.

Supplementary Figure 2

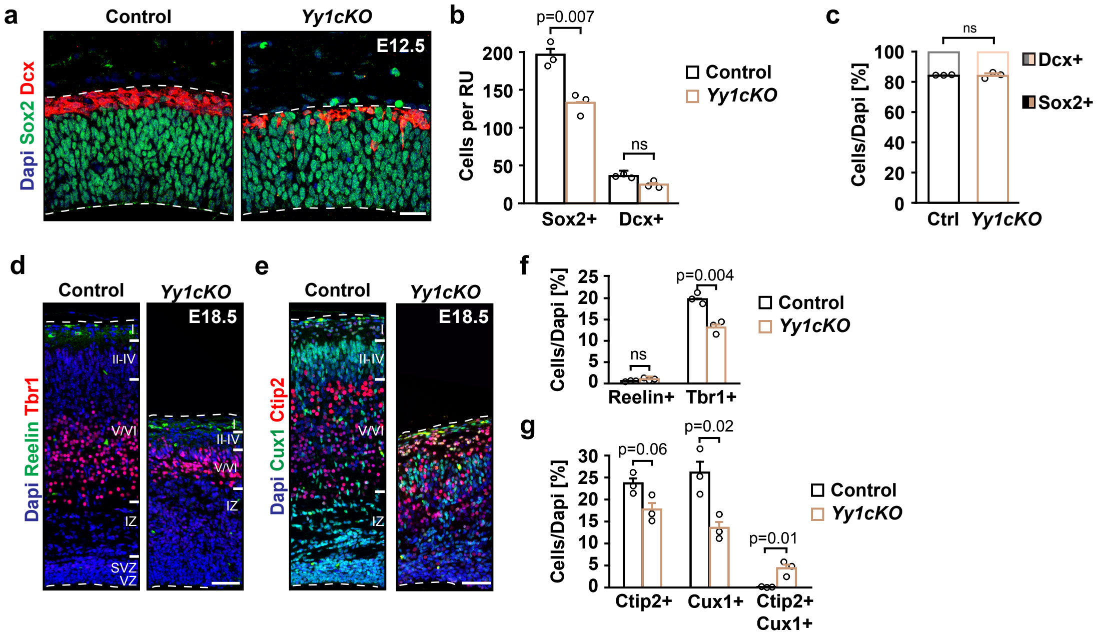

Supplementary Figure 2. Yy1 does not interfere with neuronal differentiation.

a-c) Immunostaining for Sox2+ NPCs and Dcx+ immature neurons at E12.5. Even though the absolute numbers of Sox2+ cells per radial unit (RU) are decreased upon knockout of Yy1 **(b)**, the ratio of Sox2+/Dcx+ cells does not change **(c)**.

d-g) Yy1 deletion does not affect the production Reelin+ neurons (layer I) but decreases the number Tbr1+ neurons (layer V/VI) at E18.5 **(d,f)**. Lack of Yy1 tends to decrease the number of Ctip2+ (layer V/VI) and decreases the number of Cux1+ (layer II-IV) neurons. In addition, neurons expressing both Ctip2 and Cux1 appear in Yy1 mutant embryos **(e,g)**.

Ventricular zone (VZ), subventricular zone (SVZ), intermediate zone (IZ), neuronal layers (I, II-IV, V/VI) are indicated where distinguishable.

Nuclei are counterstained with Dapi. Scale bars represent 25 μm **(a,d,e).** Comparisons were performed using the 2-tailed unpaired Student's t-test. Data are the mean ± standard deviation. * $p < 0.05$. ns = not significant.

Supplementary Figure 3

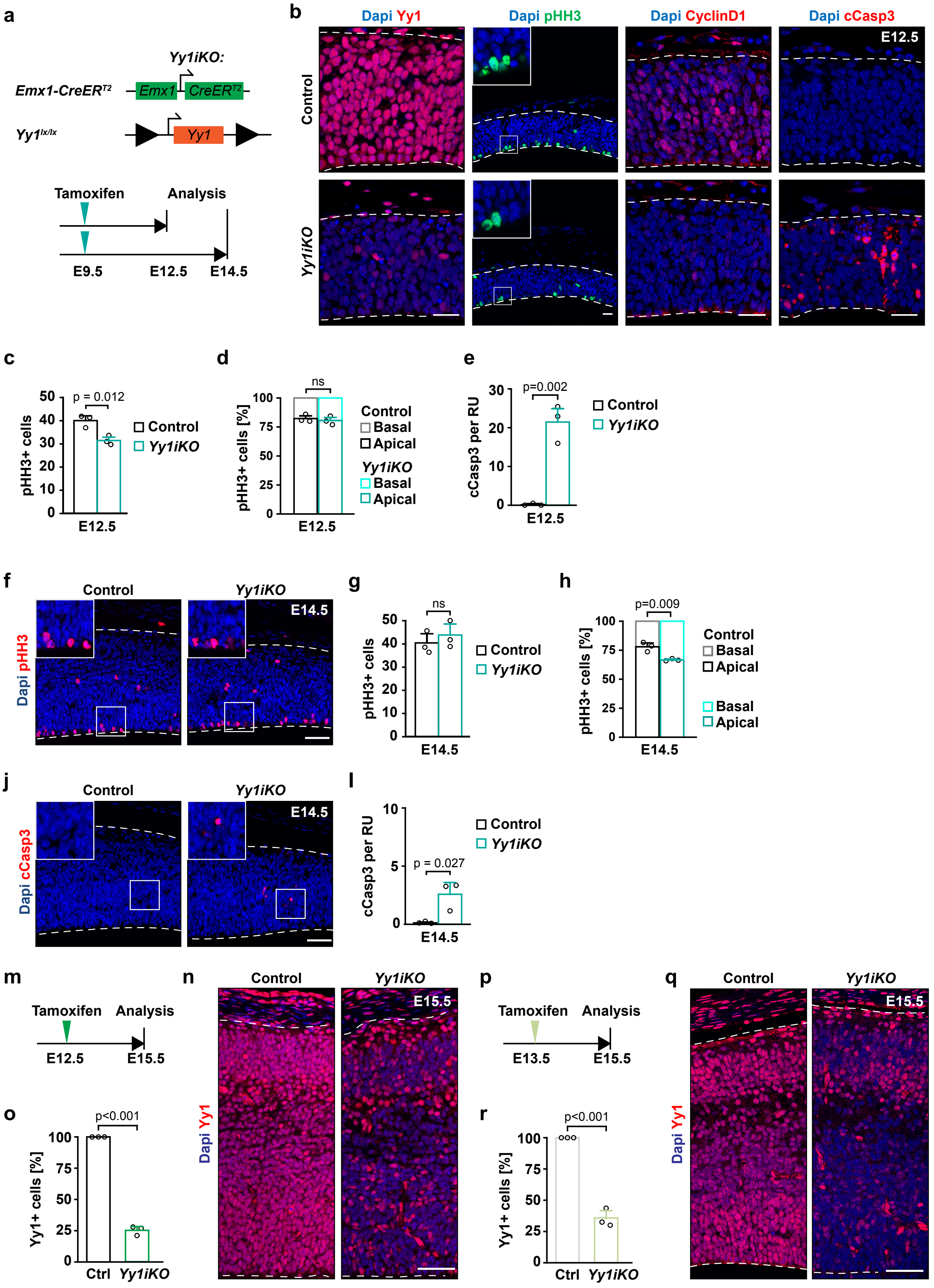

Supplementary Figure 3: Efficient ablation of *Yy1* by *Emx1CreER^{T2}* recapitulates phenotype upon recombination with *Emx1Cre*.

a) Genotype of mice and experimental strategy used to induce ablation of *Yy1*.

b-l) Tamoxifen-induced ablation of *Yy1* at E9.5 recapitulates the stage-dependent phenotype of *Yy1cKO* embryos. In *Yy1iKO* embryos, the number of pHH3+ cells decrease and the number of cCasp3+ cells increase at E12.5 **(b,c,e)**. By E14.5, this phenotype is ameliorated: the number of pHH3+ cells is comparable to controls and only a few cCasp3+ are found in *Yy1iKO* embryos **(f-l)**.

m) Experimental strategy to ablate *Yy1* at E12.5 in *Yy1iKO* embryos.

n,o) Immunostaining for Yy1 and quantification at E15.5 to analyze the recombination efficiency upon tamoxifen-induced knockout of *Yy1* at E12.5.

p) Experimental strategy to ablate *Yy1* at E13.5 *Yy1iKO* embryos.

q,r) Immunostaining for Yy1 and quantification at E15.5 to analyze the recombination efficiency upon tamoxifen-induced knockout of *Yy1* at E13.5.

Nuclei are counterstained with Dapi. Scale bars represent 50 μm **(f,j)**, 20 μm **(b,n,q).** Comparisons were performed using the 2-tailed unpaired Student's t-test. Data are the mean ± standard deviation. * $p < 0.05$. ns = not significant.

Supplementary Figure 4

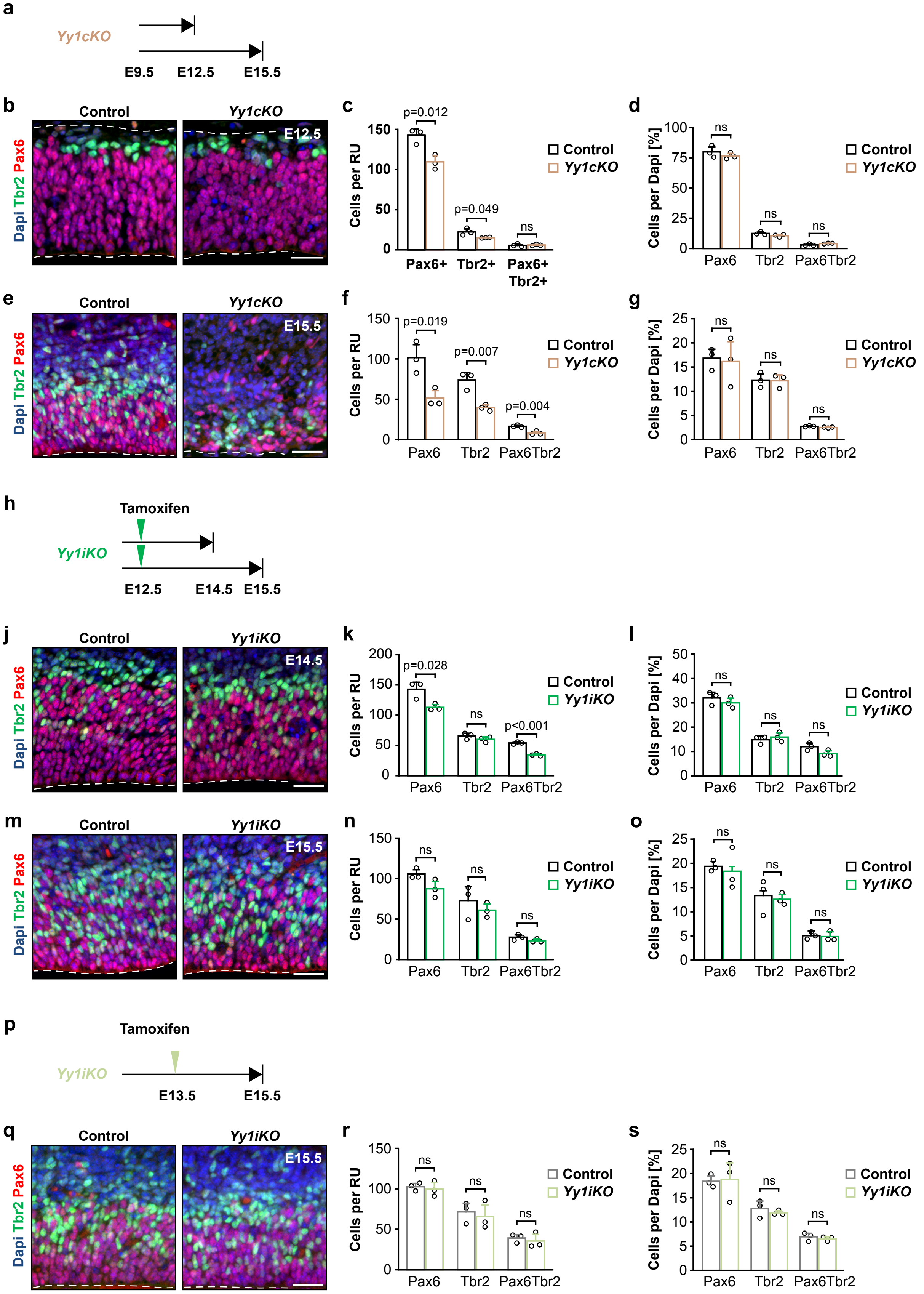

Supplementary Figure 4: Apical and intermediate progenitor cells are not differentially affected by knockout of *Yy1*.

a-g) Immunostaining for markers identifying apical progenitor (AP) cells (Pax6+), basal progenitor (BP) cells (Tbr2+) and cells transitioning from AP to BP (Pax6+Tbr2+) reveal that Yy1 does not influence the differentiation of NPCs. While the absolute numbers of Pax6+ and Tbr2+ NPCs are decreased in *Yy1cKO* embryos at E12.5 and E15.5 **(b,c,e,f)**, the ratio of AP/BP cells is not affected **(d,g)**.

h-o) TM-induced ablation of *Yy1* at E12.5 alters the number of Pax6+ cells at E14.5 but not at E15.5 **(j,k,m,n)**. The number of Tbr2+ and Pax6Tbr2+ cells and relative abundance of Pax6+, Tbr2+ and Pax6+Tbr2+ cells are not affected **(l,o)**.

p-s) TM-induced ablation of *Yy1* at E13.5 does not influence the number or ratio of Pax6+, Tbr2+ and Pax6+Tbr2+ cells at E15.5.

Nuclei are counterstained with Dapi. Scale bars represent 25 µm **(b,e,j,m,q)**. Comparisons were performed using the 2-tailed unpaired Student's t-test. Data are the mean ± standard deviation. ns = not significant.

Supplementary Figure 5

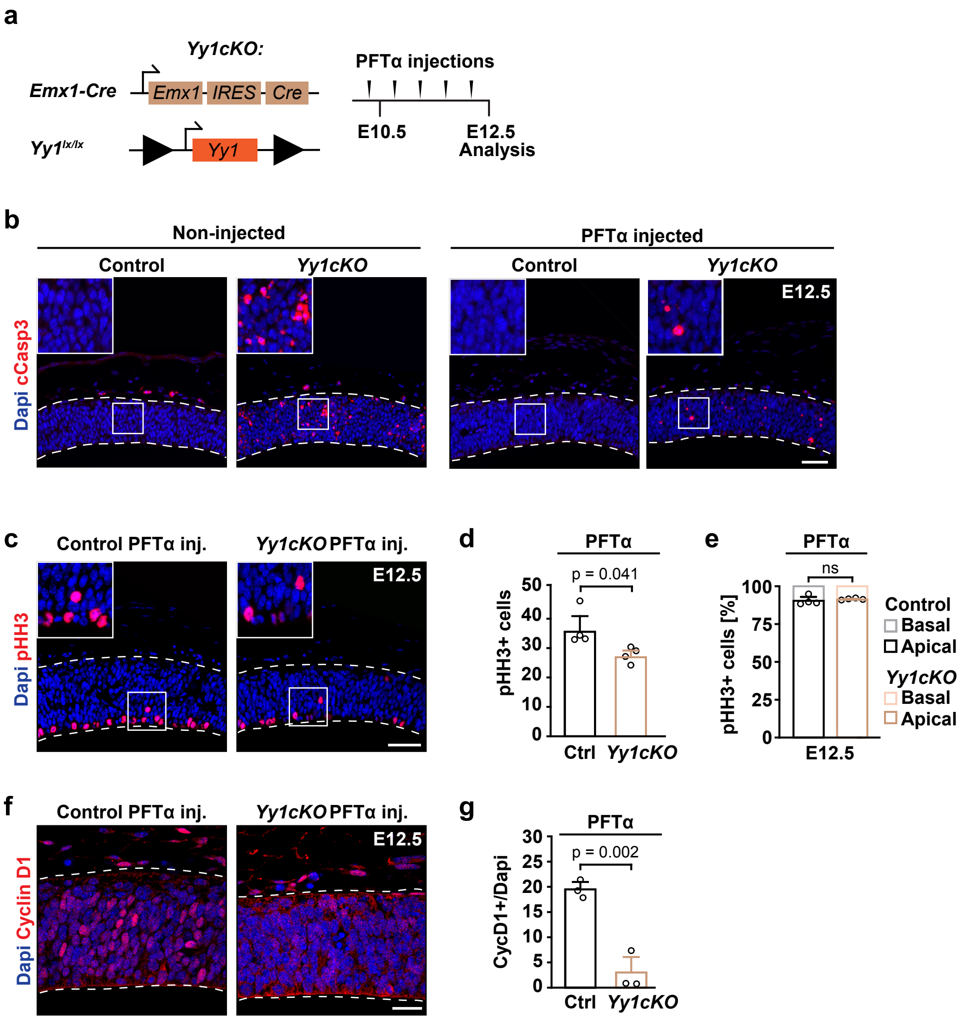

Supplementary Figure 5: Pharmacological inhibition of p53-signaling reduces cell death in Yy1cKO embryos.

a) Experimental strategy to inhibit p53 signaling by intraperitoneal injections of Pifithrin α (PFTα inj.) two times per day from E10-E12.

b) Immunostaining for cleaved Caspase 3 shows that inhibition of p53 signaling decreases the number of apoptotic cells in *Yy1cKO* embryos compared to non-injected Yy1cKO embryos.

c-e) Inhibition of p53 signaling does not restore the number of mitotic pHH3+ cells in *Yy1cKO* embryos. The numbers of pHH3+ cells are normalized to 600 µm VZ length.

f,g) Inhibition of p53 signaling does not rescue the number of CyclinD1+ cells in *Yy1cKO* embryos.

Nuclei are counterstained with Dapi. Scale bars resemble 50 µm **(b)**, 20 µm **(c,f)**. Comparisons were performed using the 2-tailed unpaired Student's t-test. Data are the mean ± standard deviation. ns = not significant.

Supplementary Figure 6

a

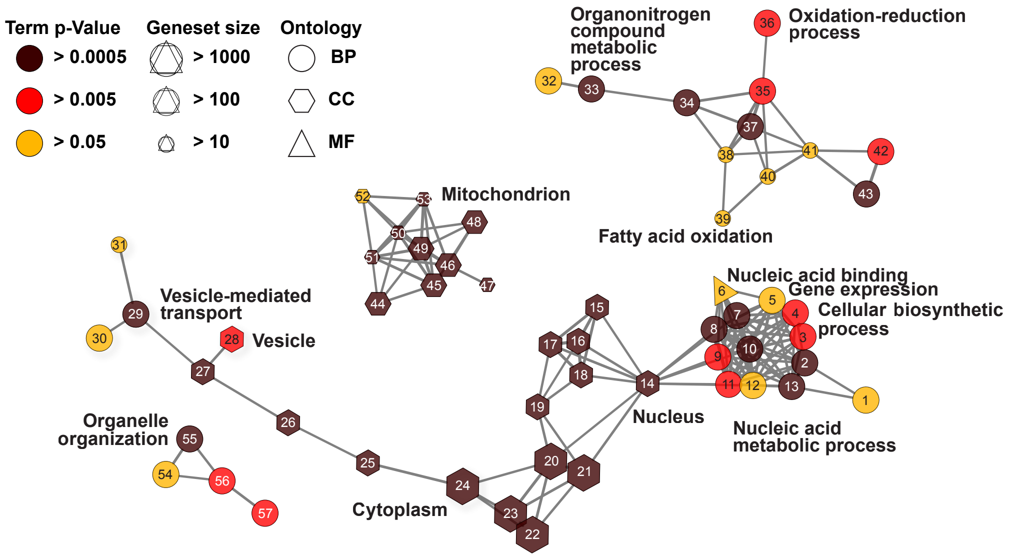

b

Node	GOTerm	GO Term ID	Term p-Value	% Associated Genes	Nr. Genes
1	macromolecule modification	GO:0043412	47.0E-3	9.64	367
2	macromolecule metabolic process	GO:0043170	210.0E-6	9.11	799
3	organic substance biosynthetic process	GO:1901576	3.2E-3	9.42	534
4	**cellular biosynthetic process**	GO:0044249	3.2E-3	9.44	526
5	**gene expression**	GO:0010467	15.0E-3	9.43	478
6	**nucleic acid binding**	GO:0003676	15.0E-3	9.70	385
7	cellular nitrogen compound metabolic process	GO:0034641	97.0E-9	9.91	599
8	organic cyclic compound metabolic process	GO:1901360	490.0E-6	9.54	543
9	cellular aromatic compound metabolic process	GO:0006725	800.0E-6	9.54	527
10	heterocycle metabolic process	GO:0046483	390.0E-6	9.60	524
11	nucleobase-containing compound metabolic process	GO:0006139	520.0E-6	9.60	514
12	**nucleic acid metabolic process**	GO:0090304	6.4E-3	9.55	458
13	cellular macromolecule metabolic process	GO:0044260	2.4E-6	9.40	751
14	**nucleus**	GO:0005634	81.0E-12	10.09	657
15	nucleoplasm	GO:0005654	100.0E-6	10.94	253
16	nuclear lumen	GO:0031981	4.0E-6	10.73	332
17	nuclear part	GO:0044428	74.0E-9	10.77	383
18	organelle lumen	GO:0043233	600.0E-15	11.46	410
19	intracellular organelle part	GO:0044446	120.0E-30	11.01	810
20	intracellular organelle	GO:0043229	70.0E-45	10.48	1170
21	intracellular membrane-bounded organelle	GO:0043231	380.0E-39	10.62	1044
22	intracellular	GO:0005622	2.6E-54	10.12	1374
23	intracellular part	GO:0044424	83.0E-48	10.10	1315
24	**cytoplasm**	GO:0005737	53.0E-36	10.45	1066
25	cytoplasmic part	GO:0044444	110.0E-27	10.96	770
26	endomembrane system	GO:0012505	2.5E-6	10.52	374
27	intracellular vesicle	GO:0097708	400.0E-6	11.49	183
28	**vesicle**	GO:0031982	720.0E-6	9.99	385
29	**vesicle-mediated transport**	GO:0016192	21.0E-6	12.50	153
30	transport	GO:0006810	17.0E-3	9.60	411
31	regulation of vesicle-mediated transport	GO:0060627	32.0E-3	13.81	62
32	cellular amide metabolic process	GO:0043603	6.3E-3	12.24	113
33	**organonitrogen compound metabolic process**	GO:1901564	83.0E-6	11.24	223
34	small molecule metabolic process	GO:0044281	8.7E-6	11.74	208
35	organic acid metabolic process	GO:0006082	580.0E-6	12.82	114
36	**oxidation-reduction process**	GO:0055114	3.6E-3	12.02	127
37	oxoacid metabolic process	GO:0043436	160.0E-6	13.30	108
38	monocarboxylic acid metabolic process	GO:0032787	29.0E-3	13.17	74
39	**fatty acid oxidation**	GO:0019395	9.7E-3	22.68	22
40	small molecule catabolic process	GO:0044282	6.2E-3	16.25	46
41	single-organism catabolic process	GO:0044712	9.7E-3	12.69	93
42	cellular catabolic process	GO:0044248	820.0E-6	11.65	162
43	organic substance catabolic process	GO:1901575	230.0E-6	11.48	190
44	organelle membrane	GO:0031090	96.0E-9	11.62	267
45	organelle envelope	GO:0031967	630.0E-9	13.48	141
46	mitochondrial part	GO:0044429	19.0E-15	16.55	144
47	mitochondrial matrix	GO:0005759	3.4E-6	18.39	57
48	**mitochondrion**	GO:0005739	680.0E-24	14.98	262
49	mitochondrial envelope	GO:0005740	58.0E-9	15.71	101
50	mitochondrial membrane	GO:0031966	170.0E-9	15.81	95
51	organelle inner membrane	GO:0019866	2.2E-6	16.35	77
52	mitochondrial membrane part	GO:0044455	42.0E-3	17.39	32
53	mitochondrial inner membrane	GO:0005743	1.0E-6	17.11	71
54	single-organism organelle organization	GO:1902589	27.0E-3	10.65	193
55	**organelle organization**	GO:0006996	210.0E-9	10.92	343
56	cellular component assembly	GO:0022607	830.0E-6	10.66	253
57	macromolecular complex subunit organization	GO:0043933	3.3E-3	10.67	229

Supplementary Figure 6. Pathways and protein localizations of genes regulated by Yy1. Related to Fig.4.

Complete labeling of the Gene Ontology (GO) term network analysis in Figure 4B including percentage of associated genes per GO term and number of genes differentially regulated in Yy1cKO embryos. Each node represents an enriched GO term (adjusted p value (Corrected with Bonferroni step down procedure) < 0.05). Nodes are interconnected when the gene overlap is >50%, based on the kappa score. BP, biological process; MF, molecular function; CC, cellular component. Bold GO terms are highlighted in the network.

Supplementary Figure 7

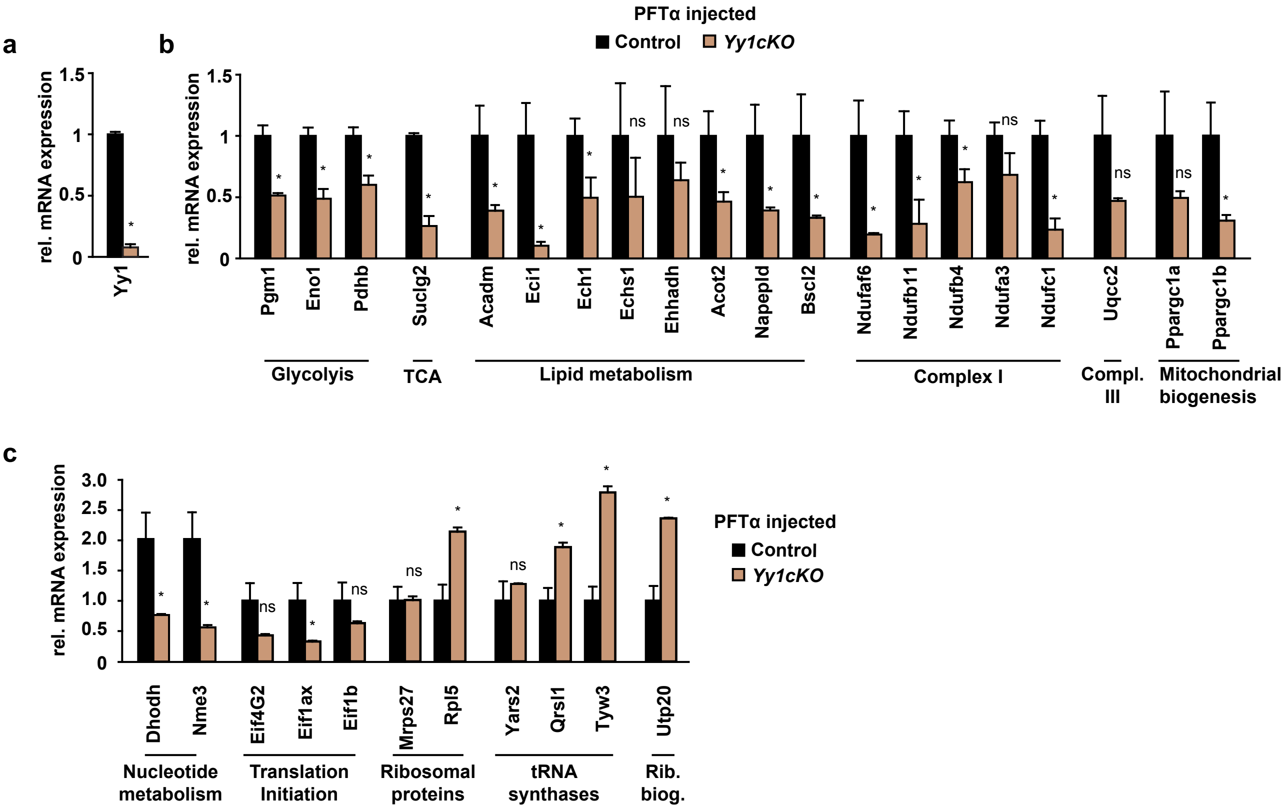

Supplementary Figure 7. Inhibition of p53 signaling does not affect expression of metabolic genes in *Yy1cKO* embryos.

a-c) qRT-PCR for Yy1-regulated genes upon inhibition of p53 signaling by injection of PFTα. Comparisons were performed using the 2-tailed unpaired Student's t-test. Data are the mean ± standard deviation. * $p < 0.05$. ns = not significant.

Supplementary Figure 8

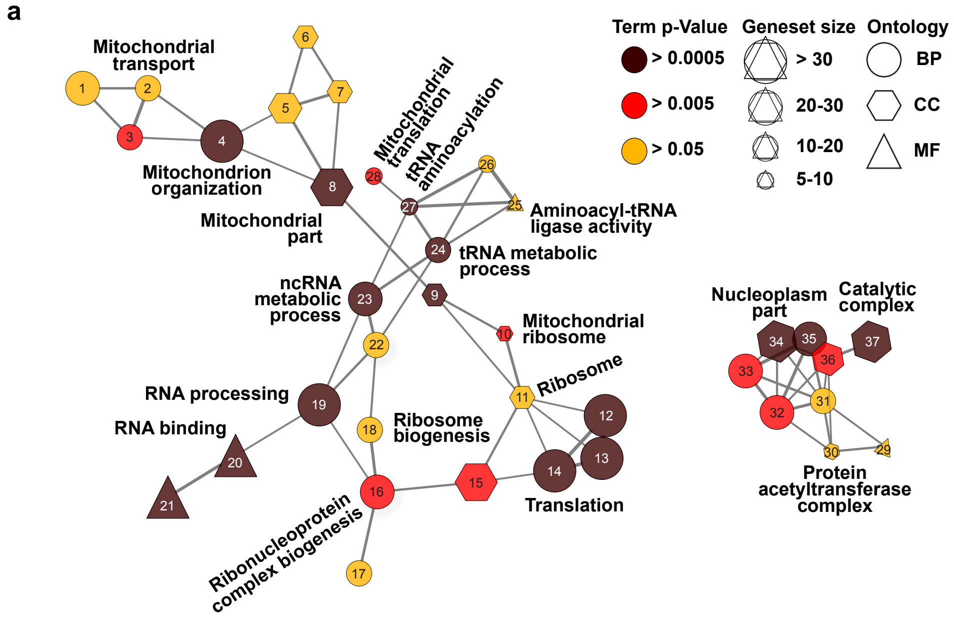

b

Node	GOTerm	GO Term ID	Term p-Value	% Associated Genes	Nr. Genes
1	protein targeting	GO:0006605	5.4E-3	4.31	24
2	**mitochondrial transport**	GO:0006839	13.0E-3	6.02	13
3	protein targeting to mitochondrion	GO:0006626	3.7E-3	8.03	11
4	**mitochondrion organization**	GO:0007005	28.0E-6	4.91	31
5	mitochondrial membrane	GO:0031966	16.0E-3	4.10	24
6	mitochondrial membrane part	GO:0044455	17.0E-3	6.71	11
7	organelle inner membrane	GO:0019866	49.0E-3	4.16	19
8	**mitochondrial part**	GO:0044429	3.3E-6	4.63	39
9	mitochondrial matrix	GO:0005759	160.0E-6	6.38	19
10	**mitochondrial ribosome**	GO:0005761	4.6E-3	11.11	8
11	**ribosome**	GO:0005840	23.0E-3	5.38	14
12	peptide metabolic process	GO:0006518	150.0E-6	4.29	34
13	amide biosynthetic process	GO:0043604	42.0E-6	4.54	34
14	**translation**	GO:0006412	5.2E-6	5.07	33
15	intracellular ribonucleoprotein complex	GO:0030529	680.0E-6	4.23	32
16	**ribonucleoprotein complex biogenesis**	GO:0022613	580.0E-6	5.43	21
17	ribonucleoprotein complex assembly	GO:0022618	20.0E-3	6.12	12
18	**ribosome biogenesis**	GO:0042254	15.0E-3	5.62	14
19	**RNA processing**	GO:0006396	21.0E-6	4.68	34
20	**RNA binding**	GO:0003723	1.1E-12	4.59	73
21	poly(A) RNA binding	GO:0044822	36.0E-12	4.98	58
22	ncRNA processing	GO:0034470	5.4E-3	5.59	16
23	**ncRNA metabolic process**	GO:0034660	10.0E-6	6.23	24
24	**tRNA metabolic process**	GO:0006399	100.0E-6	8.81	14
25	**aminoacyl-tRNA ligase activity**	GO:0004812	17.0E-3	13.04	6
26	tRNA aminoacylation for protein translation	GO:0006418	17.0E-3	13.04	6
27	**tRNA aminoacylation**	GO:0043039	260.0E-6	16.33	8
28	**mitochondrial translation**	GO:0032543	720.0E-6	14.29	8
29	N-acyltransferase activity	GO:0016410	12.0E-3	8.49	9
30	**protein acetyltransferase complex**	GO:0031248	38.0E-3	8.16	8
31	peptidyl-lysine modification	GO:0018205	17.0E-3	5.05	16
32	histone modification	GO:0016570	1.6E-3	5.06	21
33	chromatin organization	GO:0006325	920.0E-6	4.30	29
34	**nucleoplasm part**	GO:0044451	3.9E-6	4.93	35
35	chromatin modification	GO:0016568	380.0E-6	4.70	27
36	transferase complex	GO:1990234	4.0E-3	4.09	28
37	**catalytic complex**	GO:1902494	11.0E-6	4.20	43

Supplementary Figure 8. Pathways and protein localizations of genes bound by Yy1. Related to Fig.5.

Complete labeling of the Gene Ontology (GO) term network analysis in Figure 5D including percentage of associated genes per GO term and number of genes bound by Yy1 at E12.5. Each node represents an enriched GO term (adjusted p value (Corrected with Bonferroni step down procedure) < 0.05). Nodes are interconnected when the gene overlap is $>50\%$, based on the kappa score. BP, biological process; MF, molecular function; CC, cellular component. Bold GO terms are highlighted in the network.

Supplementary Figure 9

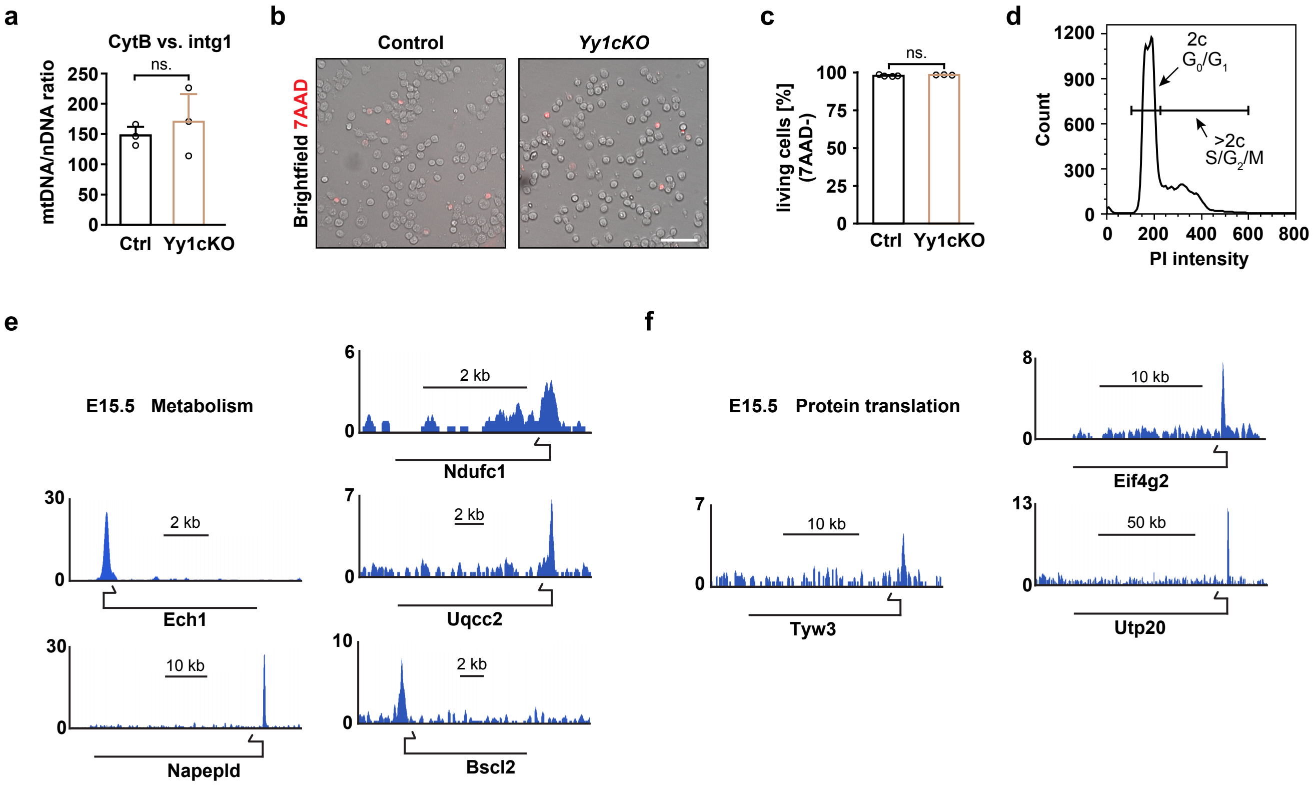

Supplementary Figure 9: Yy1cKO does not affect mitochondrial DNA content.

a) qRT-PCR for mitochondrial DNA content shows no difference between *Yy1cKO* and control cortex cells. Graph present mitochondrial (CytB) versus nuclear (intergenic region, intg1) DNA ratio.

b,c) *Yy1cKO* cells do not exhibit increased sensitivity to OCR measurement preparation procedures. The percentage of living (7AAD-negative) cells just before OCR measurement was more than 97% in control and *Yy1cKO* conditions.

d) Example of flow cytometry gating strategy to distinguish between G_0/G_1 and $S/G_2/M$ phase cells according to propidium iodide (PI) incorporation.

e,f) Genomic snapshots depicting Yy1 binding events at metabolic genes **(e)** and genes involved in protein translation **(f)** at E15.5. kb, kilo bases.

Comparisons were performed using the 2-tailed unpaired Student's t-test. Data are the mean ± standard deviation. ns = not significant.

Supplementary Figure 10

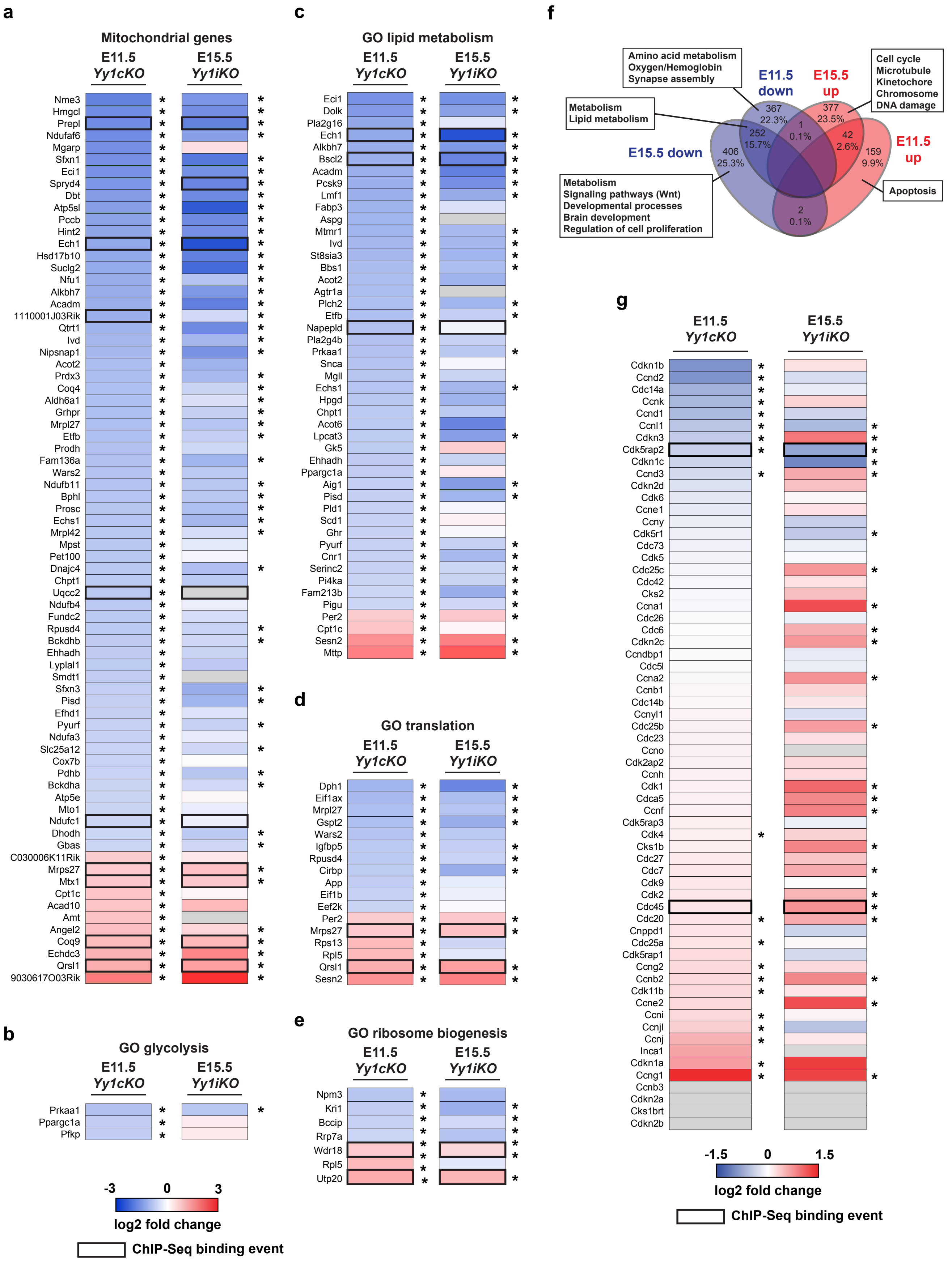

Supplementary Figure 10: Yy1 regulates similar genes at early and late cortical stages.

a-e) Expression of genes which are differentially expressed (|log2 fold change| > 0.58, $p < 0.05$, FDR < 0.05) in E11.5 *Yy1cKO* cells and their relative fold change in *Yy1iKO* Prom1+ cells at E15.5 upon TM-injection at E12.5. Gene products localized to mitochondria (MitoCarta 2.0) **(a)**, glycolytic process (GO:0006096) **(b)**, genes from the GO term lipid metabolism (GO:0044255) **(c)**, GO terms translation (GO:0006412) **(d)** and ribosome biogenesis (GO:0042254) **(e)**. Boxes indicate Yy1 binding events in E12.5 and/or E15.5 ChIP-Seq datasets. Grey color indicates genes which were not detected by RNA-Seq.

f) Comparison of up-and downregulated genes of RNA-Seq datasets E11.5 and E15.5. Relevant GO terms determined using ClueGO are indicated for the respective groups.

g) Expression of cyclins, cyclin-dependent kinases and cell-division cycle genes from GO term cell cycle (GO:0007049) in *Yy1cKO* and *Yy1iKO* vs controls at E11.5 and E15.5, respectively. Boxes indicate Yy1 binding events in E12.5 and/or E15.5 ChIP-Seq datasets. Grey color indicates genes which were not detected by RNA-Seq. Comparisons were performed using the 2-tailed unpaired Student's t-test. * $p < 0.05$.

Supplementary Figure 11

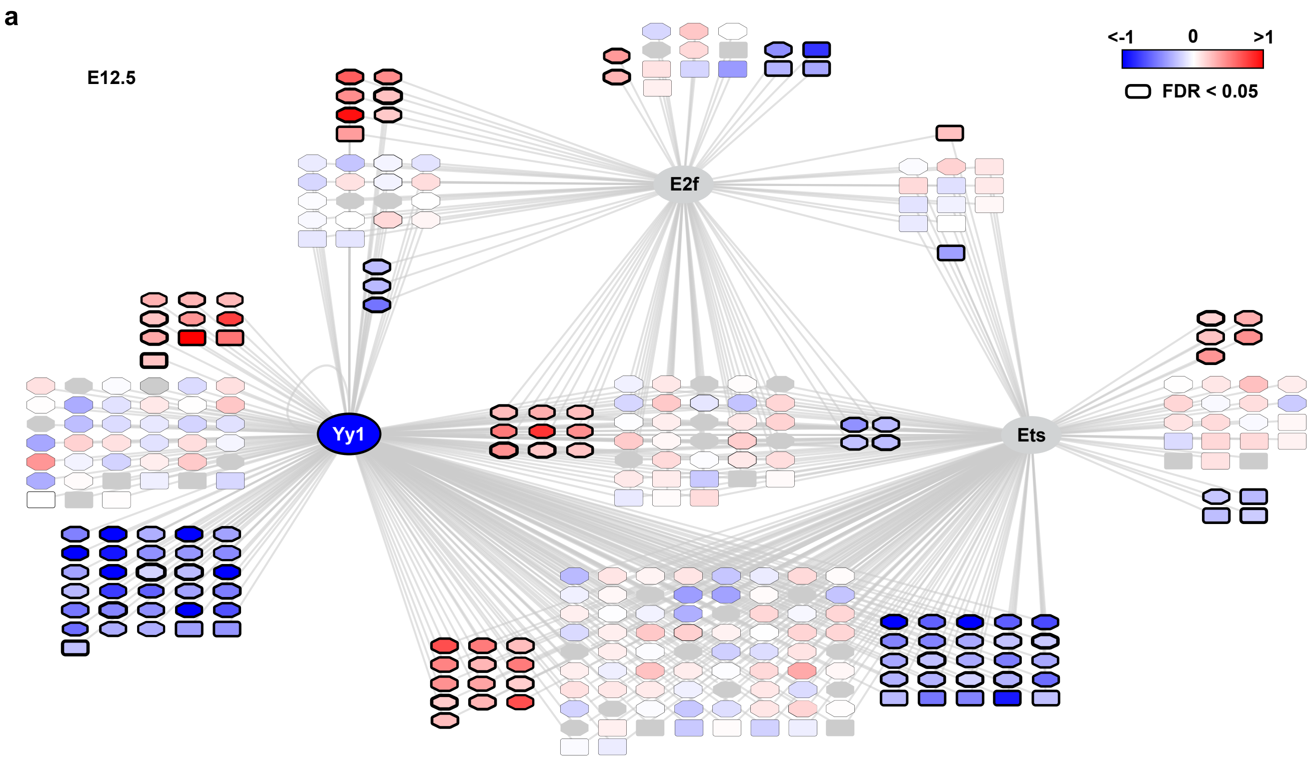

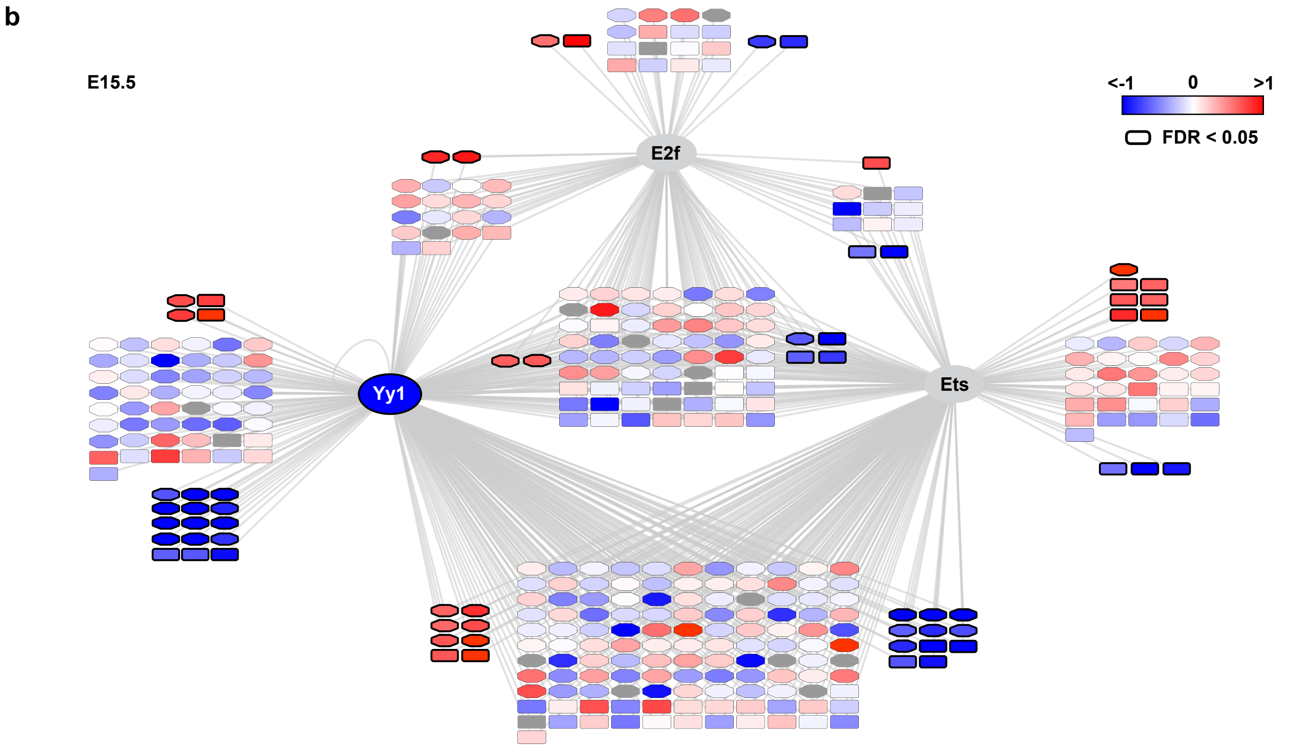

Supplementary Figure 11: Computational prediction of Yy1 co-factors.

a,b) i-cisTarget based analysis identified motifs of E2f and Ets family members as significantly enriched in Yy1 ChIP-Seq datasets at both E12.5 and E15.5 (1 ChIP-Seq replica each). Network edges connect known, annotated co-bound genes to their respective transcription factors. Note that nodes which are not connected to Yy1 illustrate newly described binding events in this study and are therefore not connected to Yy1. Hexagons indicate nodes which are found at both developmental stages, rounded rectangles are found only at one developmental stage.

Supplementary Figure 12

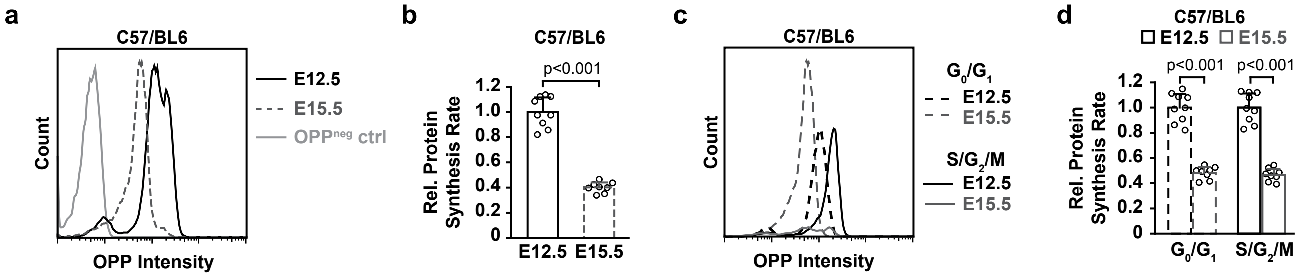

Supplementary Figure 12: Protein synthesis rate decreases along cortex development in C57/Bl6 embryos.

a-d) Protein translation rate of E12.5 and E15.5 cortical cells in C57/Bl6 embryos relative to E12.5. OPP incorporation in cortical cells in G_0G_1 (DNA content = 2c) and S/G_2/M (DNA content > 2c) phases of the cell cycle **(c,d)**. DNA content was determined using propidium iodide. Comparisons were performed using the 2-tailed unpaired Student's t-test.

SUPPLEMENTARY TABLES

Supplementary Table 1: Short interfering RNA sequences.

siRNA	Sequence	Provider
**siYy1 I**	GCGTTCGTTGAGAGCTCAAAGCTAA	Thermo Scientific
**siYy1 II**	CCGAGATCGTGGAGCTGCATGAGAT	Thermo Scientific

Supplementary Table 2: Primers for qRT-PCR

Primers	Forward 5'-3'	Reverse 5'-3'
**AcadM**	TTCGAAGACGTCAGAGTGCC	CTGCGACTGTAGGTCTGGTT
**Acot2**	AGTGCCTATGAAGGACTGAGG	TCTTACGGCACTGGGGAATG
**Acot6**	CGAGCGTCACTTCATGGCTC	AATCGATGATCCCAGGGAACTG
**Bscl2** [1]	GGCTCCTTCTACTACTCCTACA	CCGATCACGTCCACTCTT
**Dhodh**	CGGGCTTAATGGCAGAAGGA	CCGTGGCTGTCAGGTAAGAG
**Ech1**	ATTGAGAAGTGCCCCAAGCC	AGCGAATGTCACAGGCAGAA
**Echs1**	CTGTTGTCCTCAGTCCGCTG	TTCAACTGGATCAGCCCCAC
**Eci1**	TCCCTCAGCTTGGAGTGTCT	GATACCCGGGCATTCCGAC
**Ehhadh**	GCCATAGTGATCTGTGGAGCA	ACCACTGGCTTCTGGTATCG
**Eifax**	GGCTACGAGATTACCAGGACA	CTCGCCATAGGCCTTGAGAC
**Eif1b**	TTCTATCCTCTCACCGGCCT	AAGGGGTCGAAAGATTGGAGG
**Eif4G2**	ACCCACAATGGGACGTCATC	GCCTCAAACTAATCTGGCTTTTCA
**Eno1**	GGGTGGATTCGCACCTAACA	GCCAGACCTGTAGAACTCGG
**Mrps6**	GCTGCTGCTTTGAAACGTACA	AGGAAATACCCTCCTCGGCTG
**Mrps27**	GCCTCGCAGGTAAAAGATGC	TGCAAGATCAGCCAGATGGT
**Napepld** [2]	TTCTTTGCTGGGGATACTGG	GCAAGGTCAAAAGGACCAAA
**Ndufa3**	GATGGCCGGGAGAATCTCTG	GCATACTTGGTGTAGGGGCT
**Ndufaf6**	TTCAATGTGGAACTGGCTCA	TGACAGCCTTCCAGAGTTCA
**Ndufb11**	TACGCGAAGAACCCTGACTT	AGGCCATTGACTTCTCGGTA
**Ndufb4**	GGCCCCTCATCTTCTGGTATT	CAAGAATAGTCATCCTTGCCGA
**Ndufc1**	CGTAGTGCTGCGCTCGTTT	CTTCGACCGTGTTGAAGAGCAG
**Nme3**	TTTCTGCATGGAGGTTGGCA	TCACGGAACCAAAGAGCGAT
**P53**	ACCGCCGTACAGAAGAAGAA	CGGAACATCTCGAAGCGTTT
**Pdhb**	TTGCCCATTCAAGACCAGTG	GTCTGATGGTGCGCAGATTTA
**PGC1α**	ATGACCCTCCTCACACCAAAC	CTTGAGCATGTTGCGACTGC
**PGC1β**	CGCTCCAGGAGACTGAATCC	CTTGACTACTGTCTGTGAGGC
**Pgm1**	CAGTGGGACTGAGCCCAAAA	GGCACCGACTAACTCATCCA
**Qrsl1**	TCAATGGACGTGCCAGGAAT	CCAGCCAGTGTACCTAACACA
**Rpl5**	GGGTTGCTCAAAAGAAGGCA	AGCTTAACACAGAAAAAGCTGC
**Sesn2**	TAGCCTGCAGCCTCACCTAT	GATTTTGAGGTTCCGTTCCA
**Suclg2**	CGCATGGCAGAAAATCTGGG	GGGGTTCACTTCCACCTGAG

**Tbp** [3]	ACTCCTGCCACACCAGCTTC	CGAAGTGCAATGGTCTTTAGGTC
**Tyw3**	GCACAGAAGGCTCTGGAGTT	CCTTCAGAGCTGCCATCACA
**Uqcc2**	CCAGTGGACGAGACCAAACG	CCTCGGGTTCTGCAATCTGG
**UTP20**	AGGGACCCAACCTGAAGAGA	GAACAGCAGTCTCCGTCCTT
**Yars2**	GGGCGGATCAGATCAGTTGG	ATCCTCTCCAGTCAACTTGTGG
**Ywazh** [3]	ATTTCCATGTTGGGCACAGG	AAAGATCATGCGGCCCTTTT
**Yy1**	GTGGTTGAAGAGCAGATCATTGG	TTGCTTAGGGTCTGAGAGGTC
**β-Actin**	CCATCCTGCGTCTGGACCTG	GTAACAGTCCGCCTAGAAGC

References for primers used:

1. Cui, X. *et al.* Seipin ablation in mice results in severe generalized lipodystrophy. *Hum. Mol. Genet.* **20,** 3022–3030 (2011).

2. Geurts, L. *et al.* Adipose tissue NAPE-PLD controls fat mass development by altering the browning process and gut microbiota. *Nat. Commun.* **6,** 6495 (2015).

3. Alsiö, J. M., Tarchini, B., Cayouette, M. & Livesey, F. J. Ikaros promotes early-born neuronal fates in the cerebral cortex. *Proc. Natl. Acad. Sci.* **110,** E716-E725 (2013)

Supplementary Table 3: Primers for determination of mitochondrial versus genomic DNA content.

**Primers for mitochondrial vs genomic DNA**	**Forward 5‘-3‘**	**Reverse 5‘-3‘**
**Intergenic region (Genomic)**	GCTCCGGGTCCTATTCTTGT	TCTTGGTTTCCAGGAGATGC
**Mit1 (mitochondrial DNA)**	ATGGTACGGACGAACAGACG	CGATGTCTCCGATGCGGTTA
**CytB1 (mito. DNA)**	GGCTACGTCCTTCCATGAGG	TGGGATGGCTGATAGGAGGT
