# Supplementary material for: Yin Yang 1 sustains biosynthetic demands during brain development in a stage-specific manner
Source: Nat Commun. 2019 May 16;10:2192. doi: 10.1038/s41467-019-09823-5 (PMC6522535; doi:10.1038/s41467-019-09823-5)
Supplement: Supplementary file 3 — Reporting Summary [file 41467_2019_9823_MOESM3_ESM.pdf]

nature research

Corresponding author(s): Lukas Sommer

Last updated by author(s): Mar 9, 2019

Reporting Summary

Nature Research wishes to improve the reproducibility of the work that we publish. This form provides structure for consistency and transparency in reporting. For further information on Nature Research policies, see Authors & Referees and the Editorial Policy Checklist.

Statistics

For all statistical analyses, confirm that the following items are present in the figure legend, table legend, main text, or Methods section.

n/a	Confirmed	
☐	☒	The exact sample size (*n*) for each experimental group/condition, given as a discrete number and unit of measurement
☐	☒	A statement on whether measurements were taken from distinct samples or whether the same sample was measured repeatedly
☐	☒	The statistical test(s) used AND whether they are one- or two-sided<br>*Only common tests should be described solely by name; describe more complex techniques in the Methods section.*
☒	☐	A description of all covariates tested
☒	☐	A description of any assumptions or corrections, such as tests of normality and adjustment for multiple comparisons
☐	☒	A full description of the statistical parameters including central tendency (e.g. means) or other basic estimates (e.g. regression coefficient) AND variation (e.g. standard deviation) or associated estimates of uncertainty (e.g. confidence intervals)
☐	☒	For null hypothesis testing, the test statistic (e.g. *F*, *t*, *r*) with confidence intervals, effect sizes, degrees of freedom and *P* value noted<br>*Give P values as exact values whenever suitable.*
☒	☐	For Bayesian analysis, information on the choice of priors and Markov chain Monte Carlo settings
☒	☐	For hierarchical and complex designs, identification of the appropriate level for tests and full reporting of outcomes
☒	☐	Estimates of effect sizes (e.g. Cohen's *d*, Pearson's *r*), indicating how they were calculated

Our web collection on statistics for biologists contains articles on many of the points above.

Software and code

Policy information about availability of computer code

Data collection	MS Excel, Adobe Photoshop, Adobe Illustrator, GraphPad, FlowJo, Leica Imaging Software, ImageJ, Seahorse Extracellular Flux Analyzer Software, Cytoscape
Data analysis	MS Excel, Adobe Photoshop, Adobe Illustrator, GraphPad, FlowJo, Leica Imaging Software, ImageJ, Seahorse Extracellular Flux Analyzer Software, Cytoscape

For manuscripts utilizing custom algorithms or software that are central to the research but not yet described in published literature, software must be made available to editors/reviewers. We strongly encourage code deposition in a community repository (e.g. GitHub). See the Nature Research guidelines for submitting code & software for further information.

Data

Policy information about availability of data

All manuscripts must include a data availability statement. This statement should provide the following information, where applicable:

- Accession codes, unique identifiers, or web links for publicly available datasets
- A list of figures that have associated raw data
- A description of any restrictions on data availability

The sequencing datasets generated and analysed during the current study are available in the European Nucleotide Archive (https://www.ebi.ac.uk/ena) and are accessible through the accession numbers PRJEB21545, PRJEB30271 and PRJEB21635. All relevant data that support the findings of this study are available from the corresponding author upon reasonable request. Source data for all figures are provided as a Source Data file.

Field-specific reporting

Please select the one below that is the best fit for your research. If you are not sure, read the appropriate sections before making your selection.

☒ Life sciences ☐ Behavioural & social sciences ☐ Ecological, evolutionary & environmental sciences

For a reference copy of the document with all sections, see nature.com/documents/nr-reporting-summary-flat.pdf

Life sciences study design

All studies must disclose on these points even when the disclosure is negative.

Sample size	No sample-size calculations were performed.
Data exclusions	No data were excluded
Replication	Attempts to replicate the experiments were successful.
Randomization	Samples were not randomized (genotype known).
Blinding	Researchers were not blined.

Reporting for specific materials, systems and methods

We require information from authors about some types of materials, experimental systems and methods used in many studies. Here, indicate whether each material, system or method listed is relevant to your study. If you are not sure if a list item applies to your research, read the appropriate section before selecting a response.

Materials & experimental systems

n/a	Involved in the study	
☐	☒	Antibodies
☒	☐	Eukaryotic cell lines
☒	☐	Palaeontology
☐	☒	Animals and other organisms
☒	☐	Human research participants
☒	☐	Clinical data

Methods

n/a	Involved in the study	
☐	☒	ChIP-seq
☐	☒	Flow cytometry
☒	☐	MRI-based neuroimaging

Antibodies

Antibodies used	anti-cleaved caspase 3 (rabbit Cell signaling 9661, 1:300), anti-Ctip2 (rat Abcam ab18465, 1:200), anti-Cyclin B1 (rabbit Santa Cruz sc-752, 1:200), anti-Cyclin D1 (mouse Santa Cruz sc-450, 1:50), anti-Dcx (guinea pig Millipore ab2253, 1:300), anti-p53 (rabbit Santa Cruz sc-6243, 1:50; mouse Cell Signaling, 1:300), anti-Pax6 (mouse DSHB, 1:50 and rabbit Covance PRB-278P, 1:200), anti-phospho Histone 3 (mouse PH3 Cell Signaling 9706, 1:300), anti-Reelin (mouse Novus Biological NB600-1081, 1:100), anti-Sox2 (rabbit Chemicon AB5603, 1:100 and mouse R&D MAB2018), anti-Tbr1 (rabbit Abcam ab31940, 1:200), anti-Tbr2 (rabbit Chemicon AB9618, 1:200), anti-Yy1 (rabbit sc1703 and mouse sc-7341, both Santa Cruz, 1:100). anti-β-actin (mouse Sigma a5316, 1:10000), anti-Histone H3 (mouse Cell Signaling, 1:1000), (rat CD133 1:250, 14-1331-80 eBioscience; Dylight 488 anti-rat 1:500, 112-486-072, Thermo Scientific)
Validation	Antibodies are commercially available (see suppliers above).

Animals and other organisms

Policy information about studies involving animals; ARRIVE guidelines recommended for reporting animal research

Laboratory animals	Mice. Males and females from the following genotypes: Emx1-Cre Yy1lx/wt, Emx1-Cre Yy1wt/wt, Yy1lx/lx, and Emx1-CreERT2 Yy1lx/wt, Emx1-Cre ERT2 Yy1wt/wt, Emx1-Cre Yy1lx/lx, Emx1-CreERT2 Yy1lx/lx, Emx1-Cre Yy1lx/lx Trp53 lx/lx, Emx1-Cre Yy1lx/wt Trp53lx/lx, Wild-type C57/Bl6, NMRI or Swiss mice (Janvier, France)
Wild animals	Study did not involve wild animals.
Field-collected samples	No field collected samples.

Ethics oversight	No ethical approval was required. All animal experiments were conducted in accordance with Swiss guidelines and approved by the Veterinary Office of the Canton of Zurich, Switzerland.

Note that full information on the approval of the study protocol must also be provided in the manuscript.

ChIP-seq

Data deposition

☒ Confirm that both raw and final processed data have been deposited in a public database such as GEO.

☐ Confirm that you have deposited or provided access to graph files (e.g. BED files) for the called peaks.

Data access links<br>*May remain private before publication.*	https://www.ebi.ac.uk/ena
Files in database submission	PRJEB21635
Genome browser session<br>(e.g. UCSC)	no longer applicable.

Methodology

Replicates	2 replicas per developmental stage.
Sequencing depth	Sample, Raw Reads Nb, Uniquely mapped reads, percentage (%) of uniquely mapped reads<br>E12.5 sample 1 IgG (1E12IgG), 17903356, 13282192, 74.18828068<br>E12.5 sample 1 Yy1 (1E12Yy1), 17416565, 12865587, 73.86983024<br>E12.5 sample 2 IgG (2E12IgG), 17677617, 13189328, 74.61032785<br>E12.5 sample 2 Yy1 (2E12Yy1), 16254440, 12156484, 74.78869773<br>E15.5 sample 1 IgG (1E15IgG), 19107967, 14378153, 75.24690094<br>E15.5 sample 1 Yy1 (1E15Yy1), 17708642, 13398481, 75.66069154<br>E15.5 sample 2 IgG (2E15IgG), 13669681, 9594008, 70.18457856<br>E15.5 sample 2 Yy1 (2E15Yy1), 21115234, 15437698, 73.11165957
Antibodies	rabbit anti–Yy1 antibody (sc-1703, Santa Cruz), rabbit IgG antibody (Santa Cruz).
Peak calling parameters	All sequenced reads were mapped using Bowtie 2 (http://bowtie-bio.sourceforge.net/bowtie2/index.shtml) onto the UCSC mm10 reference mouse genome. The command "findPeaks" from the HOMER tool package (http://homer.salk.edu/homer/) was used to identify enriched regions in the Yy1 immunoprecipitation experiments using the "-style = factor" option (routinely used for transcription factors with the aim of identifying the precise location of DNA-protein contact). Input samples were used as enrichment-normalization control. Peak calling parameters were adjusted as following: L = 2 (filtering based on local signal), F = 2 (fold-change in target experiment over input control). The annotation of peaks' position (i.e. the association of individual peaks to nearby annotated genes or genomic loci) was obtained by the all-in-one program called "annotatePeaks.pl".
Data quality	FDR cutoff: 0.001<br>E12_1: 432 peaks<br>E12_2: 226<br>E15_1: 383<br>E15_2: 128
Software	UCSC genome browser (http://genome.ucsc.edu)

Flow Cytometry

Plots

Confirm that:

☒ The axis labels state the marker and fluorochrome used (e.g. CD4-FITC).

☒ The axis scales are clearly visible. Include numbers along axes only for bottom left plot of group (a 'group' is an analysis of identical markers).

☒ All plots are contour plots with outliers or pseudocolor plots.

☒ A numerical value for number of cells or percentage (with statistics) is provided.

Methodology

Sample preparation	Isolation of dorsal cortex cells from mouse embryos by microdissection

Instrument	FACS Canto II, FACS Aria III
Software	FACSDIVA, FlowJo
Cell population abundance	Abundance: between 5-70%, depending on the isolated cell type.
Gating strategy	Gates were set using samples stained without OPP/EdU pulse or without primary antibodies.

☒ Tick this box to confirm that a figure exemplifying the gating strategy is provided in the Supplementary Information.
