# Supplementary material for: Yin Yang 1 sustains biosynthetic demands during brain development in a stage-specific manner
Source: Nat Commun. 2019 May 16;10:2192. doi: 10.1038/s41467-019-09823-5 (PMC6522535; doi:10.1038/s41467-019-09823-5)
Supplement: Supplementary file 4 — Description of Additional Supplementary Files [file 41467_2019_9823_MOESM4_ESM.pdf]

Description of Additional Supplementary Files

File Name: Supplementary Data 1
Description: RNA-Seq GO Analysis: ClueGO gene ontology analysis of RNA-Seq data (E11.5, Control vs. Yy1cKO) related to Figure 4 and Supplementary Figure 6

File Name: Supplementary Data 2
Description: ChIP-Seq GO Analysis: ClueGO gene ontology analysis of E12.5 ChIP-Seq data related to Figure 5 and Supplementary Figure 8

File Name: Supplementary Data 3
Description: Metabolomics: List of relative metabolite abundance in siCtrl vs siYy1 related to Figure 6.
